# The high‐risk HPV E6 proteins modify the activity of the eIF4E protein via the MEK/ERK and AKT/PKB pathways

**DOI:** 10.1002/2211-5463.12987

**Published:** 2020-11-19

**Authors:** Vicente Morales‐Garcia, Adriana Contreras‐Paredes, Eduardo Martinez‐Abundis, Nancy P. Gomez‐Crisostomo, Marcela Lizano, Fernanda Hernandez‐Landero, Erick de la Cruz‐Hernandez

**Affiliations:** ^1^ Division Academica Multidisciplinaria de Comalcalco Universidad Juarez Autonoma de Tabasco Comalcalco Mexico; ^2^ Unidad de Investigación Biomédica en Cancer Instituto Nacional de Cancerología‐ Instituto de Investigaciones Biomédicas Universidad Nacional Autónoma de México Mexico City Mexico; ^3^ Departamento de Medicina Genómica y Toxicología Ambiental Instituto de Investigaciones Biomédicas Universidad Nacional Autónoma de México Ciudad Universitaria Mexico City Mexico

**Keywords:** E6 oncoprotein, eIF4E, human papillomavirus, post‐transcriptional regulation

## Abstract

Previous studies have proposed that the human papillomavirus (HPV) E6 oncoproteins modify the transcriptional activity of eIF4E through mechanisms dependent on p53 degradation. However, the effect of these oncoproteins on pathways regulating the activity of the eIF4E protein remains poorly understood. Hence, we investigated the mechanisms whereby E6 proteins regulate the activity of the eIF4E protein and its effect on target genes. Overexpression of E6 constructs (HPV‐6, HPV‐16, HPV‐18, and HPV52) showed that E6 oncoproteins increased phosphorylation of the eIF4E protein (Serine‐209). This result was mainly mediated by phosphorylation of the 4EBP1 protein via the PI3K/AKT pathway. Additionally, the pharmacological inhibition of eIF4E phosphorylation in cervical cancer cell lines substantially reduced the protein levels of CCND1 and ODC1, indicating that E6 of the high‐risk genotypes may modify protein synthesis of the eIF4E target genes by increasing the activity of the AKT and ERK pathways.

## Graphical abstract

In this study, we show that high‐risk E6 oncogenes (HPV16, HPV‐18, and HPV52) regulate eIF4E activity through downstream targets of PI3K/AKT/mTORC1 and MEK/ERK pathways, increasing translation of cellular proteins regulated by CAP‐dependent mechanisms (CCND1 and ODC1). The pharmacological inhibition of eIF4E phosphorylation by Ribavirin highlights the utility of this approach for the treatment of cervical cancer.

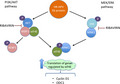

**Abbreviations**
4EBP1: eukaryotic translation initiation factor 4E‐binding protein 1
ANOVA: analysis of variance
cDNA: complementary DNA
COPE: The Committee on Publication Ethics
DMSO: dimethyl sulfoxide
EIF4E: eukaryotic translation initiation factor 4E
HR‐HPV: high‐risk human papillomavirus
MNK1/2: MAPK interacting protein kinases 1 and 2
mTORC1: mammalian target of rapamycin complex 1
ODC1: ornithine decarboxylase 1
P529: Palomid‐529
PD: PD184352
qRT–PCR: quantitative real‐time PCR
Rb: Ribavirin
S6K: ribosomal protein S6 kinase
VEGFA1: vascular endothelial growth factor A1

Persistent infection with the human papillomavirus (HPV) has been implicated in carcinogenesis in several tissues of the genitourinary tract, including the vagina, vulva, anus, and particularly, the uterine cervix [1]. The transforming activity of the oncogenic types mainly relies on the ability of E6 and E7 proteins to interact with a wide variety of host cell proteins implicated in the control of cell cycle transition, apoptosis, and cell adhesion [2]. The main mechanisms whereby E6 and E7 oncoproteins modify the normal function of protein targets are based on stimulation of their degradation via the proteasome pathway or indirect modulation of their expression at the transcriptional level [2]. These oncoproteins may also enhance the translation rate of cellular and viral proteins essential for the viral life cycle, mainly through a Cap‐dependent mechanism. The dysregulated activation of Cap‐dependent translation initiation is a strategy that many DNA and RNA viruses employ to improve the expression of viral proteins [3, 4, 5, 6, 7].

Protein synthesis activated via the Cap‐dependent mechanism is regulated by the integration of multiple signals in response to metabolic changes, stress conditions, and stimulus of cell growth and division. This interaction is translated upon the activation or inhibition of the mammalian target of rapamycin complex 1 (mTORC1), which phosphorylates two proteins, eukaryotic translation initiation factor 4E‐binding protein 1 (4EBP1) and ribosomal protein S6 kinase (S6K), that are essential for the initial stage of translation. Phosphorylation of 4EBP1 results in the release of the eukaryotic translation initiation factor 4E (eIF4E) and the subsequent formation of the eiF4F complex (i.e., the rate‐limiting step of the initial stage). However, S6 phosphorylation via the active form of S6K is important for complete assembly of the initial translation machinery [8].

The eIF4E protein regulates the translation of a select group of genes implicated in cell cycle progression (cyclin D1, CCND1), cell growth (ornithine decarboxylase, ODC1), and the synthesis of the growth factor necessary for angiogenesis (vascular endothelial growth factor A1, VEGFA1) [9]. The expression of the eIF4E protein is frequently altered in a wide variety of benign and malignant hyperproliferative disorders, including cancer [10, 11, 12]. Previous studies found that the expression of the eIF4E protein increases relative to the severity of cervical malignancy [13, 14, 15]. However, although epidemiological evidence regarding the causal relationship between HPV infection and eIF4E is scarce, the overexpression and phosphorylation of eIF4E (Serine‐209) have been associated with high‐risk HPV genotype infection [14].

The ability of HPV to enhance Cap‐dependent translation is mainly attributed to the E6 proteins via dependent (PDK1) or independent (TSC2) mechanisms of the PI3K/AKT pathway [6, 16]. In both settings, mTORC1 activation causes increased 4EBP1 and S6K phosphorylation. At the transcriptional level, eIF4E expression level is controlled by the c‐Myc proto‐oncogene, which is negatively regulated by p53 [17]. In the presence of the oncogenic E6 proteins, p53 degradation allows c‐Myc to increase the transcriptional activity of the 4E promoter [18]. The latter has been associated with the expression of the E7 oncoprotein, but experimental evidence supporting this phenomenon remains less conclusive [19]. Although increased 4EBP1 phosphorylation correlates with the elevated level of eIF4E, eIF4E phosphorylation at serine 209 depends on the MNK1/MNK2 proteins whose activity is regulated by the MEK/ERK signaling pathway [20]. Although the deregulated activity of 4E through the overactivation of the PI3K/AKT/mTORC1 axis seems to be entirely related to the expression of the E6 oncoprotein, other mechanisms contributing to eIF4E activation have not been evaluated in this context. Therefore, the aim of this study was to determine the mechanisms whereby the E6 proteins contribute to the activation of eIF4E and its effects on host proteins that are regulated by the eIF4F complex (CCND1 and ODC1).

## Materials and methods

### Cell culture and treatments

HEK293, MCF7, and cervical cancer cell lines (CasKi and HeLa) were purchased from the American Type Culture Collection. C33A and HaCat cells were kindly provided by Prof. Adriana Contreras‐Paredes (Instituto Nacional de Cancerologia, Mexico). All cell lines were grown in Dulbecco’s modified Eagle medium: Nutrient Mixture F‐12 (Thermo Fisher Scientific, Carlsbad, CA, USA) supplemented with 10% fetal bovine serum (Thermo Fisher Scientific) and 1% penicillin/streptomycin (Thermo Fisher Scientific), in a humidified environment with 5% CO_2_ at 37 °C.

Pharmacological inhibition of protein targets was achieved with the following treatments: PD184352 (10 μm, [21]); Palomid 529 (10 μm, [22]); and Ribavirin (50 μm, [23]). All drugs were dissolved in dimethyl sulfoxide (DMSO, Sigma‐Aldrich, San luis, MS, USA) and stored at −20 °C. The inhibitory effect was compared to that of control cells treated with vehicle. To evaluate protein levels, CasKi and HeLa cells were treated for 72 h, whereas for mRNA expression analysis, cells were treated for 48 h.

### Plasmid constructions

Amplification and cloning of the E6 genes from HPV6, HPV16, HPV18, and HPV52 was performed by PCR cloning of the open‐reading frames (ORFs) of these proteins between EcoRI and XhoI sites of the expression vector, pcDNA3.1 (Thermo Fisher Scientific). The same method was employed for E6/E7 constructs, but the forward primer was directed to the 5' end of E6 genes and the reverse primer to the 3′ end of E7 gene from HPV16 and HPV18. Sequences and localization of primers employed for end‐point PCR amplification are included as supporting information (Table S1). The genetic material employed for PCR amplification was obtained from cervical cancer samples previously characterized in the Laboratory of Epidemiology and Molecular Biology of Oncogenic Viruses (Prof. Marcela Lizano, Instituto Nacional de Cancerologia, Mexico). Amplification was performed with the AmpliTaq Gold DNA polymerase (Thermo Fisher Scientific), using 100 ng of extracted DNA, 2.5 mm of MgCl_2_, and 300 nm of each oligonucleotide. The obtained products and pcDNA3.1 plasmid were digested with EcoRI and XhoI enzymes and ligated with T4 DNA ligase according to the manufacturer’s conditions (Thermo Fisher Scientific). The resulting constructs were employed to transform competent bacterial cells (*E. coli*‐DH5α strain). The identity and orientation of the E6 and/or E7 genes in the clones were confirmed by DNA sequencing (Department of Synthesis and Sequencing, National Autonomous University of Mexico).

### Cell transfection

HaCat, HEK293, and MCF7 cells were transfected with the K2 transfection system (liposomes), according to the manufacturer’s standard protocol (Biontex Laboratories, Munich, Germany). Four micrograms of each E6 (6E6, E616, 18E6, and 52E6) or E6/E7 (16E6/E7 and 18E6/E7) construct was used for transfections of 1.0 × 10^4^ cells in a 6‐well plate; control cells were transfected with empty vector (pcDNA3.1). Geneticin treatment (Sigma‐Aldrich, Saint Louis, MO, USA) was added after 24 h of transfection at a concentration of 800 μg·mL^−1^. The generated cell clones were initially analyzed using end‐point PCR to verify the integrity of the transfected genes (Fig. S1). Additionally, the expression rate of the E6 and E7 mRNA genes was evaluated using qRT–PCR [24]. Therefore, only cells with similar levels of mRNA expression for the transfected genes were employed for subsequent evaluations (Figs S2 and S3).

### RNA isolation and qRT–PCR

Total RNA was isolated with the PureLink RNA Kit (Thermo Fisher Scientific), and its purity was assessed by spectrophotometric analysis (NanoDrop 2000c, Thermo Fisher Scientific). cDNA synthesis was carried out with the SuperScript III First‐Strand Synthesis SuperMix (Thermo Fisher Scientific). PCRs were carried out with QuantiNova SYBR green PCR kit (Qiagen, Hilden, Germany), supplemented with 0.35 nm of each primer set, and analyzed in a Rotor‐Gene Q thermocycler (Qiagen). Amplification was carried out for 35 cycles (94 °C for 15 s and 60 °C for 50 s) after an initial denaturation step of 95 °C for 5 min. Sequences of primers employed to assess gene expression are included as supporting information (Table S2). Data were analyzed using the $2^{‐\Delta \Delta C_{T}}$ method and reported as the fold change in gene expression normalized with respect to GAPDH expression and compared to control cells. All reactions were performed in triplicate.

### Protein extraction and immunoblot evaluation

Total proteins were extracted with RIPA buffer (Thermo Fisher Scientific), supplemented with 1 mm phenylmethylsulfonyl fluoride (Sigma‐Aldrich), and 1× protease inhibitor cocktail (Sigma‐Aldrich). Proteins (50 μg) were resolved via SDS/PAGE and analyzed by western blot using the following antibodies: pRb (IF‐8), p53 (DO1), Myc (SC‐40), ERK2 (SC‐1647), p‐ERK (Tyr‐204) (SC‐7383), AKT1/2/3 (SC‐55523), and p‐AKT (Ser‐473) (SC‐7985‐R) obtained from Santa Cruz Biotechnology (Dallas, TX, USA). CCND1 (AB16663), GAPDH (AB8245), ODC1 (AB66067), P‐eIF4E (Ser‐209) (AB76256), 4EBP1 (AB2606), p‐4EBP1 (Thr‐70) (AB75831), and MNK1 (AB89223) were purchased from Abcam (Cambridge, UK). Antibody‐immobilized membranes were incubated with a corresponding horseradish peroxidase‐conjugated secondary antibody for 2 h. Finally, the immunoreactive proteins were detected using an enhanced chemiluminescent substrate (Millipore, Burlington, MA, USA) and imaged through a gel documentation system (ImageQuant LAS 500; GE Healthcare Life Science, Björkgatan, Sweden). Furthermore, the expression of proteins was quantified by measuring the density of bands using imagej software (National Institutes of Health, Bethesda, MD, USA).

### Cytotoxicity assay

Cells were seeded into 96‐well microplates (Corning, NY, USA) at 3 × 10^3^ cells per well into 0.1 mL of complete medium. After 24 h, cells were treated for 72 h with a two‐fold serial dilution of PD184352, Palomid 529, or Ribavirin with a concentration range of 100–0.97 μm. The cytotoxicity of the drugs was measured using the XTT assay according to the manufacturer’s instructions (Roche, Mannheim, Germany). The effect of the drugs was compared to control cells treated with DMSO. All assays were performed in triplicate. The IC_50_ values were calculated using a nonlinear regression curve in graphpad prism 8.0 software (GraphPad Software, San Diego, CA, USA).

### Statistical analysis

Statistical difference between groups was compared with one‐way analysis of variance (ANOVA) followed by Dunnett’s *post hoc* test. A *P*‐value < 0.05 was considered statistically significant.

### Ethical approval

This study was designed according to international standards for research publication (COPE) and registered (protocol no. UJAT‐20160006) and approved by the Institutional Review Board of Juarez Autonomous University of Tabasco.

## Results

### High‐risk human papillomavirus E6 proteins increase phosphorylation of the eIF4E protein

**Fig. 1:**
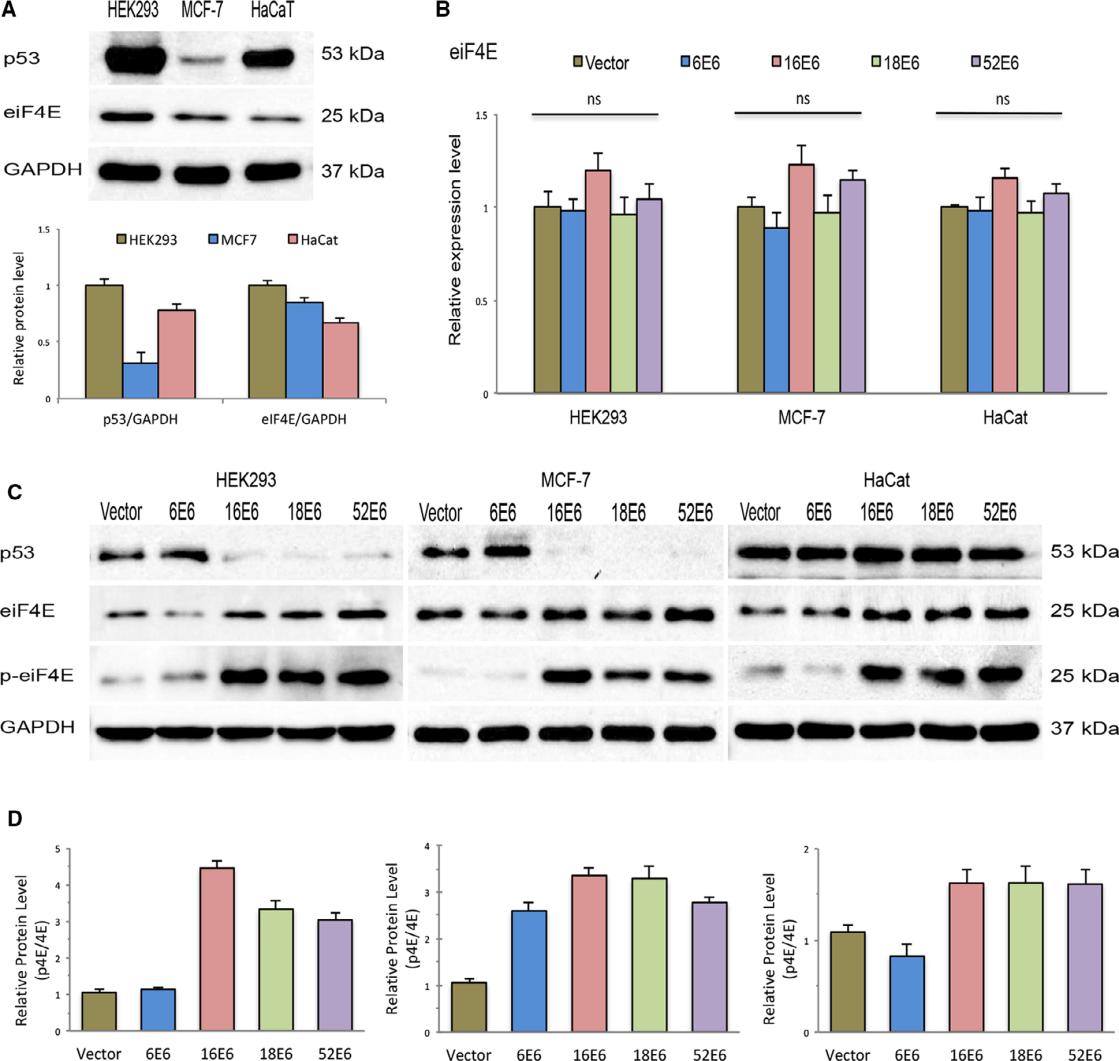
Oncogenic E6 proteins increase eIF4E phosphorylation (Serine‐209). (A) Steady‐state levels of p53, and the total and phosphorylated (Ser‐209) forms of the eIF4E protein were evaluated via immunoblot assays in HEK293 (p53: WT), MCF7 (p53: WT), and HaCat (p53: mutated) cells. Band intensity was quantified by the densitometric analysis using imagej software. At least three experiments were performed for analysis. Differences were compared by one‐way ANOVA. (B) Expression level of eIF4E mRNA in stable cells transfected with different E6 constructs (HPV‐6 E6, HPV‐16 E6, HPV‐18 E6, and HPV‐52 E6) was evaluated by real‐time RT–PCR. Control cells were transfected with empty vector (vector). Values represent mean ± SD of at least three experiments. Differences between groups were compared by one‐way ANOVA with Dunnett’s *post hoc* test (*P > 0.05; ns: no significance). (C) Total lysates of HEK293, HaCat, and MCF7 cells transfected with the different E6 constructs were resolved by SDS/PAGE and immunoblotted with antibodies against p53, total and phosphorylated forms of eIF4E proteins. GAPDH was employed as a loading control for RT–PCR and western blot analysis. D, Relationship between total and phosphorylated levels of the eIF4E protein in transfected cells was evaluated by densitometric analysis. At least three experiments were performed for the evaluation.

Previous studies have suggested that the increased levels of eIF4E during cervical carcinogenesis cause an increase in the rate of synthesis of the early oncoproteins of high‐risk HPV (HR‐HPV) [14, 25]. This finding is related to the indirect effect of E6 oncoproteins on the transcriptional activation of the eIF4E promoter via the stimulation of p53 degradation. c‐Myc therefore remains free to bind to the eIF4E promoter, enhancing transcription of the eIF4E mRNA [15]. To determine whether E6 expression modifies the transcription of eIF4E, we aimed to establish cell lines stably expressing E6 from low‐risk (HPV6) and high‐risk genotypes (HPV16 and HPV18, and HPV52). Three cell lines were selected based on the p53‐status. HEK293 and MCF‐7 had wild‐type p53, whereas HaCat expressed a mutant form of p53. Immunoblot assays prior to transfection with the E6 constructs showed that compared to HEK293 cells, MCF‐7 and HaCat cells maintained significantly lower levels of the p53 and eIF4E proteins (Fig. 1A). RT–‐qPCR evaluation of transfected cells showed that the expression of oncogenic (HPV16, HPV18, and HPV52) and nononcogenic E6 (HPV6) did not significantly modify the expression of the eIF4E transcript. This finding was independent of the cellular context of the cell lines used, as similar results were obtained in all cells (Fig. 1B). Although transcription of eIF4E was not altered by E6 expression, the effect of E6 proteins on eIF4E protein levels was evaluated. Initially, the ability of the oncogenic E6 proteins to promote p53 degradation was confirmed in cell lines harboring wild‐type p53 (HEK293 and MCF‐7). As shown in Fig. 1C, p53 was markedly reduced in cells expressing the oncogenic E6 proteins (16E6, 18E6, and 52E6), whereas cells transfected with the nononcogenic E6 protein (E66) maintained levels similar to those of control cells; this result was only observed in HEK293 and MCF‐7 cells as p53 levels remained unchanged in HaCat cells transfected with the oncogenic E6 genes. However, E6 expression, mainly of the high‐risk genotypes, caused a substantial increase in the levels of total eIF4E compared to control cells; this was evident in all cell lines evaluated (Fig. 1C). As the activation of eIF4E is tightly associated with Serine‐209 (Ser‐209) phosphorylation, its phosphorylation levels were also investigated in transfected cells. Based on the western blot results, cells harboring E6 of genotypes 16, 18, and 52 had higher levels of eIF4E phosphorylation compared to cells expressing E66 and control cells (Fig. 1C). Densitometric analysis showed that phosphorylation of eIF4E had a notable correlation with the levels of total protein observed in cells expressing the oncogenic E6 genes (Fig. 1D). These results suggest that the E6 proteins of genotypes 16, 18, and 52 modify eIF4E level by augmenting its phosphorylation at ser‐209, which has been previously shown to remarkably increase the half‐life of the protein [26]. In addition, the effect of the E6 oncoproteins on the eIF4E protein was independent of the indirect regulation of p53 at the transcriptional level.

### Coexpression of E7 does not enhance phosphorylation of eIF4E caused by E6 oncoproteins

**Fig. 2:**
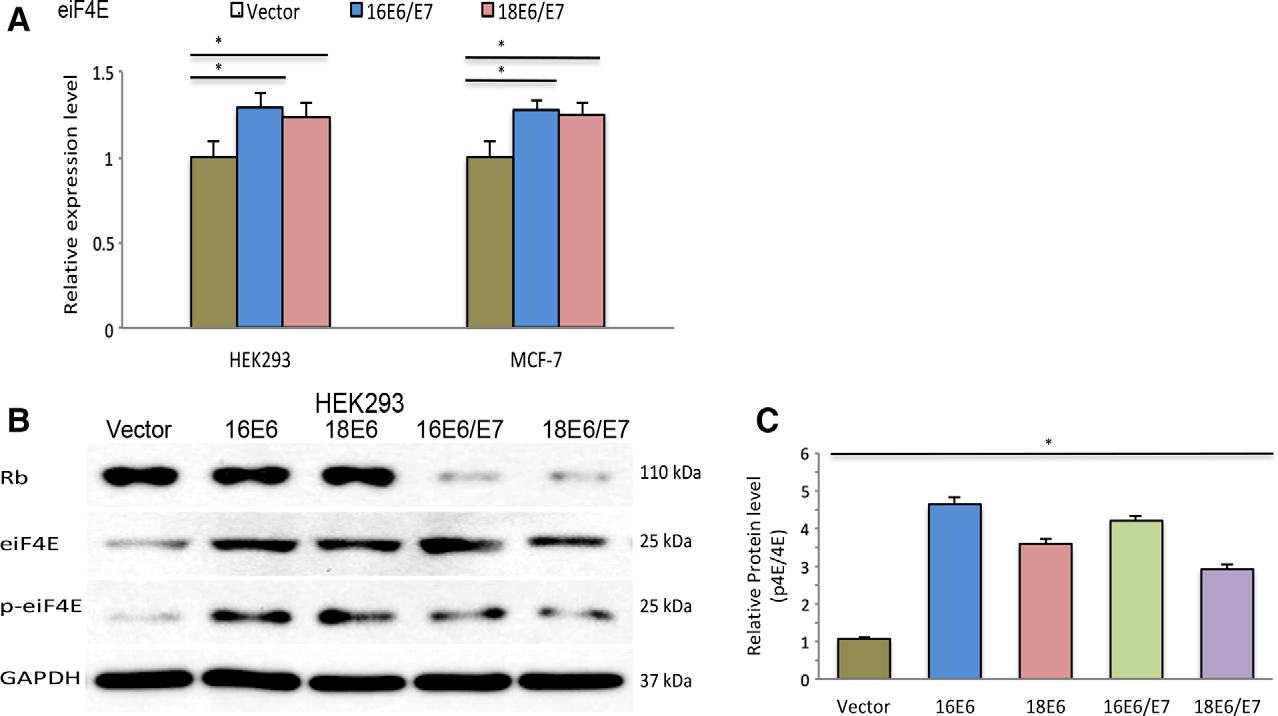
Coexpression of E7 does not enhance eIF4E phosphorylation in cells transfected with the oncogenic E6 genes. (A) Real‐time quantitative PCR results of eIF4E mRNA in stable cell lines expressing E6 or E6/E7 genes. Values represent mean ± SD of at least three experiments. Differences between groups were compared by one‐way ANOVA with Dunnett’s *post hoc* test (*P < 0.05; ns: no significance). GAPDH was employed as a loading control for RT–PCR and western blot analysis. (B) Expression of the Rb protein, and the total and phosphorylated forms of the eIF4E protein were evaluated by western blot in HEK293 cells transfected with E6 or E6/E7 ORFs from HPV‐16 and HPV‐18. (C) Relationship between total and phosphorylated levels of the eIF4E protein in HEK293 cells transfected with E6 or E6/E7 ORFs from HPV‐16 and HPV‐18 was evaluated by densitometric analysis using imagej software. At least three experiments were performed for the evaluation.

To determine whether the coexpression of E6/E7 genes may change the transcriptional activity of eIF4E mRNA, HEK293 and MCF7 cells were transfected with plasmids containing E6/E7 genes of HPV16 and HPV18. As shown in Fig. 2A, coexpression of the bicistronic mRNA containing E6 and E7 genes slightly increased the levels of eIF4E mRNA (16E6/E7: 1.3‐fold induction in HEK293 and 1.2‐fold in MCF‐7; 18E6/E7: 1.2‐fold in HEK293 and 1.2‐fold in MCF‐7) compared to cells transfected with the empty vector. To evaluate the activity of E7 in transfected HEK293 cells, pRB protein levels were evaluated by western blot analysis. As expected, cells harboring the E6/E7 genes presented a reduced level of pRB compared to cells with the E6 gene or control cells (Fig. 2B). Although cells coexpressing E6 and E7 genes showed higher levels of eIF4E phosphorylation compared to control cells, there were no remarkable differences relative to cells expressing the E6 gene alone (Fig. 2B). According to densitometric evaluation, the proportion of phosphorylated eIF4E decreases relative to the total levels in cells expressing the E6 and E7 genes (Fig. 2C). These results indicate that although E7 slightly increased the transcription of eIF4E, this upregulation did not enhance the ability of the E6 oncoproteins to activate eIF4E phosphorylation.

### E6 oncoproteins increase eIF4E phosphorylation via the AKT/PKB and ERK pathways

**Fig. 3:**
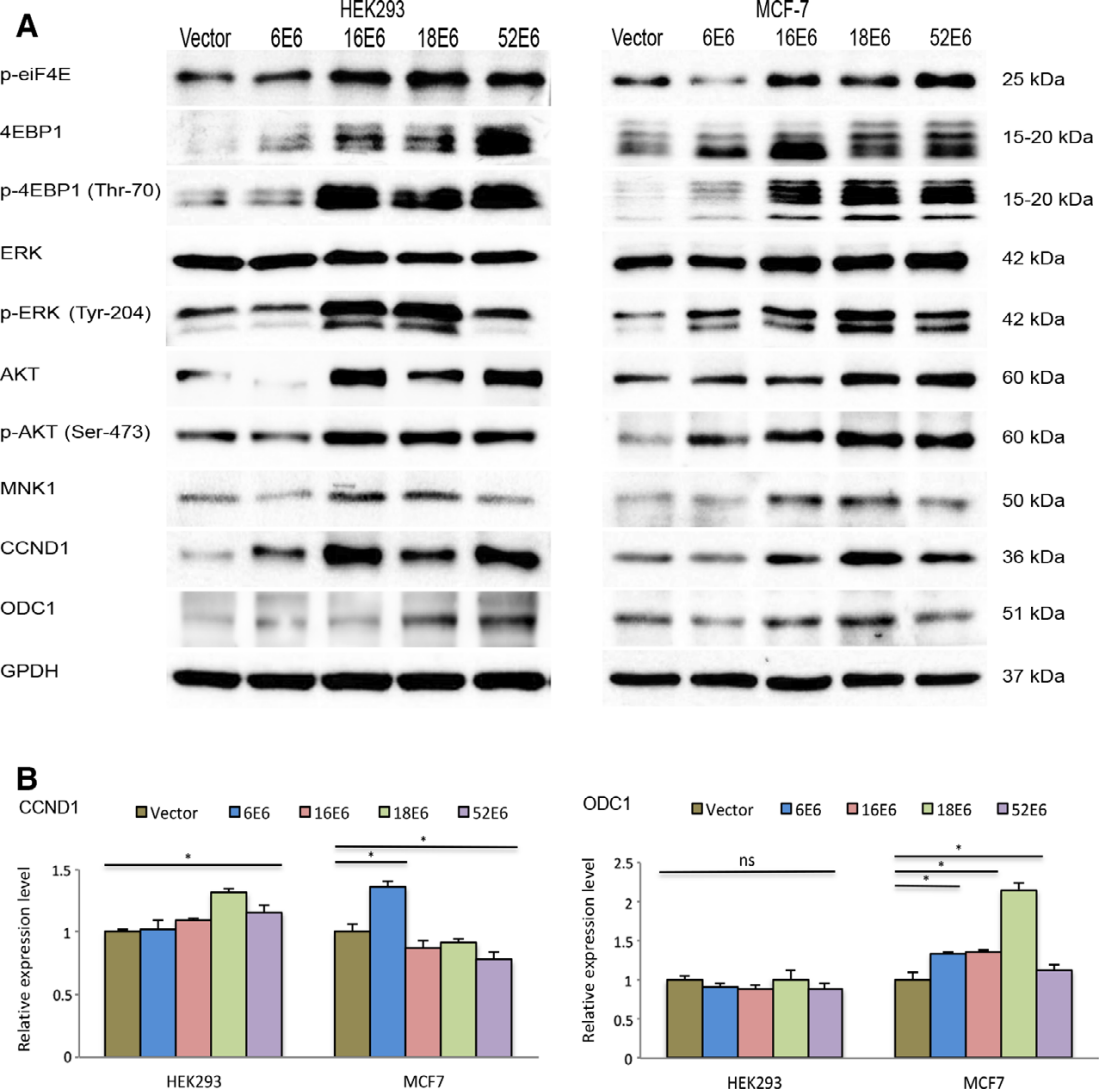
Expression of the oncogenic E6 proteins increases eIF4E phosphorylation by activating the AKT/PKB and MEK/ERK pathways. (A) Protein levels of total and phosphorylated forms of AKT and ERK, as well as the downstream targets of eIF4E gene (CCND1 and ODC1), were evaluated in HEK293 and MCF7 cells expressing E6 genes. (B) mRNA expression levels of CCND1 and ODC1 were analyzed by real‐time PCR using GAPDH to normalize mRNA levels within each sample. Values represent mean ± SD of at least three experiments. Differences between groups were compared by one‐way ANOVA with Dunnett’s *post hoc* test (*P > 0.05; ns: no significance).

The phosphorylation of eIF4E (Ser‐209) is mainly associated with two mechanisms: the inhibition of 4EBP1 via the AKT/mTORC1 axis and activation of MNK1/2 (MAPK interacting protein kinases 1 and 2) via the ERK pathway [27]. Previous studies had found that the increased activity of mTORC1 in cells expressing HPV16 E6 significantly alters the phosphorylation of 4EBP1 and S6K proteins [6]. Based on these findings, we investigated whether the increased phosphorylation of eIF4E observed in cells expressing the oncogenic E6 genes correlated with the phosphorylation level of 4EBP1. Consistent with the published results [6], the phosphorylated form of 4EBP1 (Thr‐70) was remarkably increased in cells harboring E6 genes of genotypes 16, 18, and 52. Conversely, cells transfected with 6E6 showed a slight increase in 4EBP1 phosphorylation compared to control cells (Fig. 3A). As the regulation of 4EBP1, either by mTORC1 or mTORC2, requires AKT phosphorylation at threonine residue 308 (Thr‐308) and serine residue 473 (Ser‐473), respectively [6], we sought to investigate the phosphorylation of AKT (Ser‐473). Concurrent with the phosphorylation levels of 4EBP1, cells expressing the oncogenic E6 genes showed higher levels of phosphorylated AKT. Besides the phosphorylation of 4EBP1 via the AKT pathway, contribution of the ERK pathway to eIF4E regulation was also evaluated. Because ERK phosphorylation at tyrosine residue 204 (Tyr‐204) is directly associated with phosphorylation of eIF4E [28], we focused on evaluating this modification in transfected cells. Among the E6 genes analyzed, cells transfected with 16E6 and 18E6 showed higher ERK phosphorylation, which markedly correlated with total levels of MNK1, the protein kinase responsible for eIF4E phosphorylation (Fig. 3A). This effect was also evident in cells transfected with the 52E6 construct, although only in a minor proportion (Fig. 3A). These results support the proposal that the high‐risk E6 genes (HPV16, HPV18, and HPV52) increase the phosphorylation of 4EBP1 not only via AKT activation by the mTORC2 complex but also through ERK phosphorylation and the subsequent activation of MNK1.

Moreover, to evaluate whether the phosphorylation levels of eIF4E detected in cells expressing E6 oncoproteins modify the protein synthesis of eIF4E targets, protein levels of CCDN1 and ODC1 were evaluated. As shown in Fig. 3A, the expression profile of CCDN1 and ODC1 proteins maintains remarkable similarities with the phosphorylation status of eIF4E. Higher levels of CCDN1 and ODC1 proteins were observed in HEK293 and MCF‐7 cells expressing 16E6, 18E6, and 52E6, which correlate with the increased phosphorylation of eIF4E. For CCND1, this upregulation was not related to modifications at the transcriptional level. This is because the expression level of CCND1 mRNA was slightly increased in cells transfected with high‐risk E6 genes (Fig. 3B). Conversely, 18E6 expression increased (i.e., up to 2‐fold) the transcription of ODC1 in MCF‐7 cells; however, no significant changes were observed in HEK293 cells. Based on these results, we propose that the increase in CCND1 and ODC1 proteins observed in cells expressing 16E6, 18E6, and 52E6 may be related to the higher rate of protein synthesis via the upregulated activity of the eIF4E protein.

### Pharmacological inhibition of eIF4E phosphorylation reduces the protein levels of CCND1 and ODC1 in cervical cancer cell lines

**Fig. 4:**
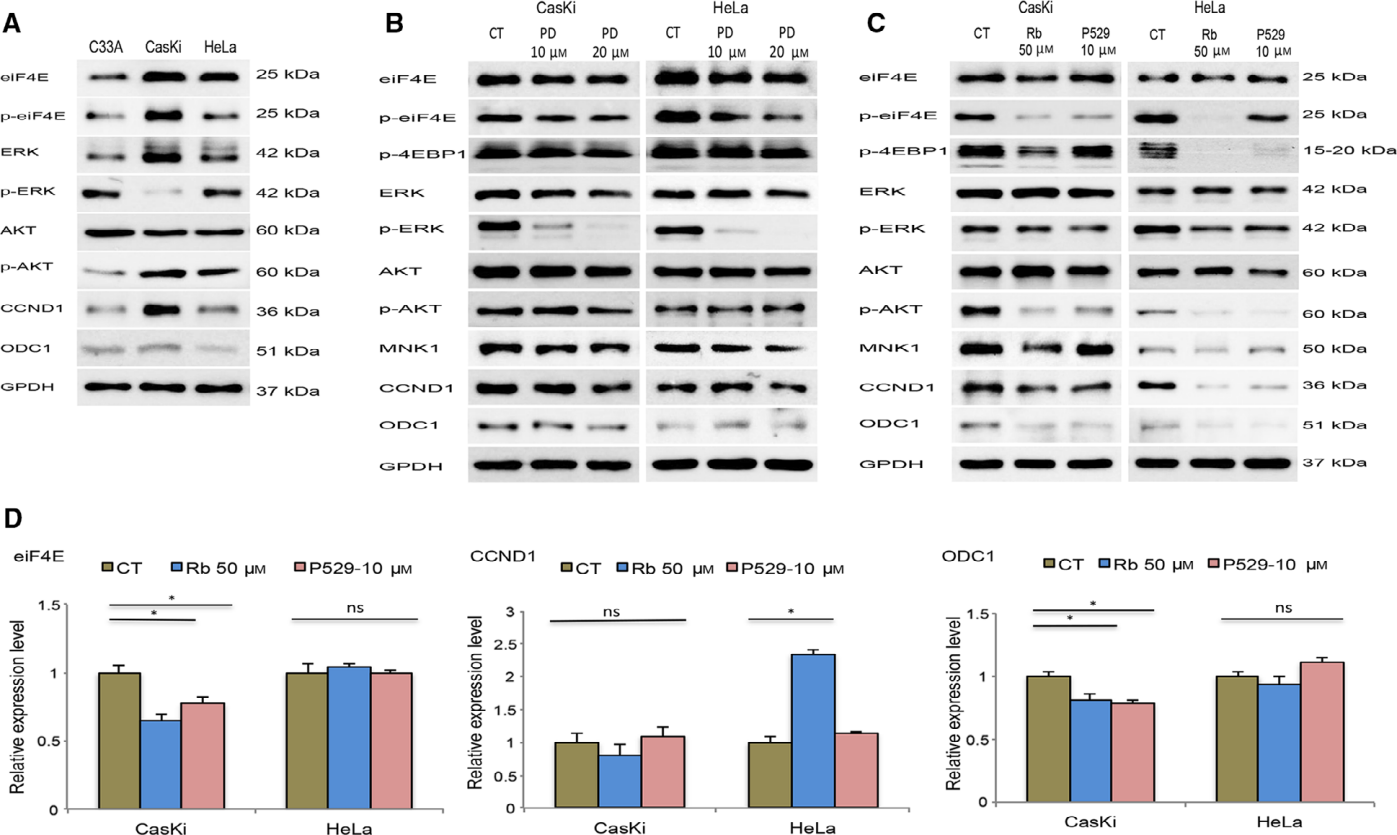
(A) Pharmacological inhibition of the AKT/PKB and ERK pathways reduces eIF4E phosphorylation and translation of the target mRNA of the eIF4F complex in cervical cancer cell lines. (A) Steady‐state levels of the phosphorylated forms of eIF4E (Ser‐209), ERK (Tyr‐204), and AKT (Ser‐473) were evaluated in C33A (HPV negative), CasKi (HPV16), and HeLa (HPV18) cell lines by immunoblot assays. Caski and HeLa cells were treated with the indicated concentrations of PD184352 (B), Palomid‐529, and Ribavirin (C) over 48 h, while control cells (CT) were incubated with DMSO. Cell lysates were resolved by SDS/PAGE and immunoblotted with antibodies against the phosphorylated form of eIF4E (Ser‐209), ERK (Tyr‐204), AKT (Ser‐473), 4EBP1 (Thr‐70), and total levels of CCND1 and ODC1 as indicated. (D) Real‐time quantitative PCR of eIF4E, CCND1, and ODC1 was performed in Caski and HeLa cells treated with Ribavirin (Rb) and Palomid 529 (P529) for 48 h. Values represent mean ± SD of at least three experiments. GAPDH was employed as a loading control for qRT–PCR and western blot assays. Differences between groups were compared by one‐way ANOVA with Dunnett’s *post hoc* test (*P > 0.05; ns: no significance).

**Fig. 5:**
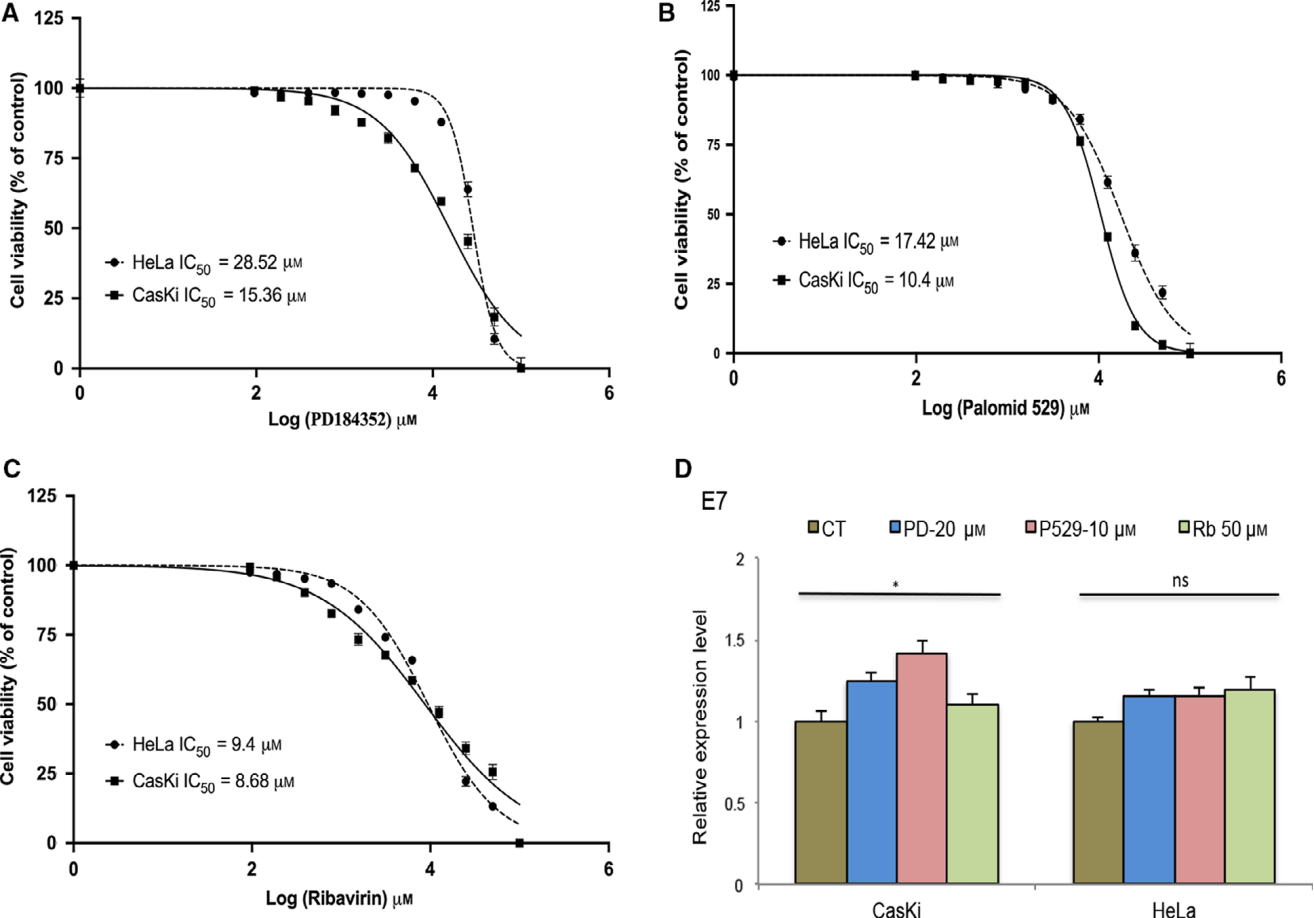
Determination of the IC50 values in CasKi and HeLa cells treated with PD184352, Palomid‐529, and Ribavirin. Cells were seeded in 96‐well plates and treated with two‐fold serial dilutions (100–0.095 µm) of PD184352 (A), Palomid‐529 (B), and Ribavirin (C) for 72 h. Following treatment, the percentage of cell survival was determined by XTT assay. The IC50 value of each drug was determined by fitting a sigmoidal dose–response curve to the data, using the GraphPad Prism 8.0 program. The *X*‐axis in each graph is presented as log_10_ values, and the data are plotted as the mean ± SD. (D), Real‐time quantitative PCR of E7 mRNA was performed in Caski and HeLa cells treated with PD184352 (PD), Palomid 529 (P529) and Ribavirin (Rb) for 48 h. Values represent mean ± SD of at least three experiments. GAPDH was employed as a loading control for RT–PCR. Differences between groups were compared by one‐way ANOVA with Dunnett’s *post hoc* test (*P > 0.05; ns: no significance).

We proceeded to investigate whether the altered regulation of CCND1 and ODC1 proteins by eIF4E was present in the cervical cancer cells. First, a panel of cervical cancer cell lines was screened to evaluate the status of upstream proteins implicated in eIF4E regulation: two HPV‐positive (CasKi and HeLa) and one HPV‐negative (C33A) cell lines. Immunoblot analysis of whole‐cell lysate revealed that eIF4E phosphorylation was notoriously higher in HPV‐positive cells (Fig. 4A). Consistent with results obtained in cells ectopically expressing high‐risk E6 genes (16E6, 18E6, and 52E6), a positive correlation was observed between AKT and eIF4E phosphorylation and levels of CCND1 and ODC1 proteins (Fig. 4A). To elucidate the role played by AKT and ERK in eIF4E activation, HPV‐positive cervical cancer cells were treated with PD184352 (MEK1/2 inhibitor [29]), Palomid‐529 (mTORC1/mTORC2 inhibitor [22]), and Ribavirin (a dual inhibitor of the PI3K/AKT and MEK/ERK pathways [23]). As shown in Fig. 4B, the pharmacological inhibition of ERK phosphorylation by PD184352 slightly reduced the levels of MNK1. Consequently, eIF4E phosphorylation was scarcely reduced. In addition, there were no meaningful changes in AKT and 4EBP1 phosphorylation. AKT inhibition by Palomid‐529 remarkably reduced 4EBP1 phosphorylation in both cell lines. Therefore, the activation of eIF4E and protein synthesis of CCND1 and ODC1 were affected (Fig. 4C). These results were complemented by the unexpected reduction in ERK phosphorylation mainly in HeLa cells. The contribution of the PI3K/AKT and MEK/ERK pathways to eIF4E activation by 4EBP1 and MNK1, respectively, was also found in cells treated with Ribavirin. Western blot data confirmed that the blockage of AKT and ERK phosphorylation by Ribavirin leads to a greater inhibition of eIF4E phosphorylation and a lower synthesis rate for the CCND1 and ODC1 proteins compared to the control and Palomid‐529‐treated cells (Fig. 4C). This result was not related to an effect at transcriptional level because qRT–PCR data indicated that neither Palomid‐529 nor Ribavirin significantly decreased the mRNA expression of CCND1 and ODC1 in CasKi and HeLa cells. Indeed, we observed some induction of CCND1 expression in HeLa cells treated with Ribavirin despite the reduction in protein levels (Fig. 4D). Such findings indicate that the aberrant activation of eIF4E, that is mainly observed in HPV‐positive cervical cancer cells, depends on the upregulation of the PI3K/AKT and MEK/ERK pathways on downstream effectors (4EBP1 and MNK1), thereby affecting the rate of translation of CCND1 and ODC1 mRNA. Additionally, we evaluated the cytotoxic effect of PD184352, Palomid‐529, and Ribavirin on CasKi and HeLa cells. The IC50 values obtained through XTT assays showed that Ribavirin (Fig. 5C) exhibited higher cytotoxic effects in comparison with the treatment with PD184352 (Fig. 5A) or Palomid‐529 (Fig. 5B). This effect was not related to the downregulation of HPV oncogenes related to stimulation of cell proliferation because neither Ribavirin nor PD184352 or Palomid‐529 significantly reduced the rate of transcription of E7 mRNA (Fig. 5D). The greater inhibition of Ribavirin on cervical cancer cell proliferation correlated notably with the synergistic effect of blocking both PI3K/AKT and MEK/ERK pathways unlike single inhibition showed by PD184352 and Palomid‐529.

## Discussion

Previous studies have suggested that the aberrant activation of the eIF4F complex is implicated in the initiation and promotion of cervical carcinogenesis‐mediated by HPV. Although the relationship between HPV infection and activation of the eIF4F complex is not completely understood, histopathological evidence indicates the involvement of eIF4E alteration in the progression of cervical cancer [13, 14]. In this study, we investigated the mechanisms through which E6 proteins of low‐risk (6E6) and high‐risk (16E6, 18E6, and 52E6) genotypes can modify the activity of the eIF4E protein and their possible effects on the downstream targets of the eIF4F complex, CCND1, and ODC1.

Consistent with other DNA viruses (e.g., adenovirus, simian virus 40, and herpes simplex virus‐1), HPV can deregulate Cap‐dependent translation machinery to favor the synthesis of viral proteins whose rate of synthesis changes according to the different phases of the viral replication. The differentiation of cervical cancer cells that are HPV‐positive augments the translation of E7 mRNA via the phosphorylation of 4EBP1 and eIF4E [7]. The ability of HPV to stimulate Cap‐dependent translation is mainly related to the expression of the E6 and E7 genes.

Changes in the eIF4E protein observed in cells expressing the E6 genes of high‐risk genotypes (16E6, 18E6, and 52E6) were mainly related to the increased level of eIF4E phosphorylation (Fig. 1). This finding was less evident in cells expressing E6 of HPV6 and was in agreement with previous studies that indicated that although most of the E6 proteins from the mucosotropic genotypes may enhance the Cap‐dependent translation, the rate of translation in the presence of nononcogenic types is notably reduced [30]. This behavior is related to structural differences in the E6 proteins (e.g., LXXL binding domains), which determines the proper interaction with cellular proteins that activate the translation machinery.

In contrast to the results published by Wang *et al*. [15], our findings did not reveal that the increased levels of eIF4E in cells expressing E6 oncoproteins may be the result of the transcriptional activation of eIF4E (Fig. 1). Indeed, although the coexpression of E6 and E7 slightly augmented the transcription of eIF4E (~ 20%), its phosphorylation was notably reduced relative to those cells expressing E6 alone (Fig. 2). According to the proposal by Pang *et al*. [19], Rb degradation induced by the E7 oncoproteins allows the E2F transcription factor to enhance the transcriptional activity of the c‐Myc promoter and the rate of eIF4E transcription. Recently, Strickland and Vande [31] showed that the expression of E7 reduces AKT phosphorylation (Thr‐308), inhibiting the phosphorylation of the ribosomal protein, S6, and ultimately promoting the activation of the Cap‐independent mechanisms via internal ribosomal entry sites (IRES) for protein synthesis. Such findings are consistent with the reduction in AKT phosphorylation in cells coexpressing the E6 and E7 genes (Fig. 3B). These results indicate that differential expression of the E6 and E7 genes could modulate the rate of protein synthesis via Cap‐dependent or independent mechanisms.

Some studies have shown that E6 genes of high‐risk genotypes (HPV16 and HPV18) increase the phosphorylation of S6K and 4EBP1 via the PI3K/AKT/mTORC1 axis [6, 30]. Hence, the phosphorylation of 4EBP1 is commonly related to eIF4E activation and the assembly of the translation initiation complex. Based on our results, the E6 proteins of genotypes 16, 18, and 52 increase the phosphorylation of AKT (Ser‐473) and 4EBP1 (Thr‐70) at amino acid residues that are mainly related to mTORC1 activation (Fig. 3A). The expression of the E6 oncoproteins also increased the phosphorylation of ERK (Tyr‐204), thereby enhancing MNK1 activation, which was associated with the higher phosphorylation level of the eIF4E protein (Fig. 3A). Consequently, cells expressing 16E6, 18E6, or 52E6 showed higher levels of the CCND1 and OCD1 proteins (Fig. 3A), which are proteins whose translation mainly occurs via Cap‐dependent mechanisms.

The pharmacological inhibition of the PI3K/AKT/mTORC1 and MEK/ERK pathways underlined the contribution of both mechanisms to eIF4E phosphorylation in cervical cancer cell lines (Fig. 4). Dual inhibition of the AKT/mTORC1 and MEK/ERK pathways by Ribavirin served as the best option to reduce eIF4E phosphorylation and consequent modification of the post‐transcriptional regulation of CCND1 and OCD1 (Fig. 4B). Ribavirin has shown an inhibitory activity over several molecular targets implicated in the development of hematological and solid tumors (e.g., EZH2, IMPDH, MYC) [32, 33, 34]; the activity over AKT/mTORC1 and MEK/ERK pathways, in addition to the inhibitory effect on cell growth (Fig. 5C), support its utility as a promising option for treatment of tumors, including cervical cancer. Further, because eIF4E phosphorylation is related to chemotherapy resistance in several gynecological malignancies, particularly DNA‐damaging agents [35, 36], concomitant treatment with pharmacological inhibitors of eIF4E could meaningfully improve the survival rate of patients with relapsed cervical cancer exhibiting chemotherapy resistance.

It is worth mentioning that, although our model was based on the overexpression of HPV E6 and E7 oncogenes that could be interpreted as a limitation of the study, the clinical significance of the results was supported by the reproducibility observed in cervical cancer cell lines assays.

Our results demonstrate that the increased phosphorylation of eIF4E observed primarily in cells transfected with high‐risk E6 oncogenes (HPV16, HPV‐18, and HPV52), as well as in HPV‐positive cervical cancer cell lines (CasKi and HeLa), is mainly related to the activation of 4EBP1 and MNK1 proteins, which are downstream targets of PI3K/AKT/mTORC1 and MEK/ERK pathways, respectively. Consequently, higher levels of cellular proteins regulated by CAP‐dependent mechanisms (CCND1 and ODC1) are observed in the presence of E6 oncoproteins. The pharmacological inhibition of eIF4E phosphorylation through blocking of PI3K/AKT/mTORC1 and MEK/ERK highlights the utility of this approach for the treatment of cervical cancer.

## Conflict of interest

The authors declare no conflict of interest.

## Author contributions

ACP, EDH, and ML involved in conception and design of the study. VMG, FHL, and EDH carried out the experiments. EMA and NPGC analyzed the data. EMA, ACP, ML, and EDH wrote the manuscript.

## Supporting information


**Fig S1.** Amplification of E6 genes was evaluated in cells transfected with different E6 constructs (HPV‐6 E6, HPV‐16 E6, HPV‐18 E6, and HPV‐52 E6) by end‐point PCR. Cells transfected with empty vector (Control cells) and a non‐template control (NTC) were included in each PCR reaction. As a loading control, a fragment of the beta‐globin gene was amplified. PCR products were resolved by electrophoresis in a 1% agarose gels stained with SYBR green.

**Fig S2.** Expression level of E6 mRNA in HEK293 (A), MCF7 (B), and HaCat (C) cells transfected with different E6 constructs (HPV‐6 E6, HPV‐16 E6, HPV‐18 E6, and HPV‐52 E6) was evaluated by RT‐qPCR. Expression levels of cells transfected with 16E6, 18E6, and 52E6 were compared with cells transfected with 6E6; at least two different clones obtained after geneticin selection were evaluated. Data of gene of interest were normalized with respect to GAPDH expression. Values represent mean ± SD of at least three experiments. Differences between groups were compared by one‐way ANOVA with Dunnett’s post hoc test (*p> 0.05; ns: no significance).

**Fig S3.** Expression levels of E6 and E7 genes in HEK293 cells transfected with different E6/E7 constructs were evaluated by RT‐qPCR. Expression levels of E6 and E7 genes were compared between cells transfected with 16E6/E7 and 18E6/E7; at least two different clones obtained after geneticin selection were evaluated. Data of gene of interest were normalized with respect to GAPDH expression. Values represent mean ± SD of at least three experiments. Differences between groups were compared by one‐way ANOVA with Dunnett’s post hoc test (*p> 0.05; ns: no significance).

**Table S1.** Primers sequences employed for end‐point PCR amplification.
**Table S2.** Primer sequences for evaluation of gene expression by real‐time PCR.

## Data Availability

Data will be available from the corresponding author upon reasonable request.
