# Supplementary figures and images for: The high‐risk HPV E6 proteins modify the activity of the eIF4E protein via the MEK/ERK and AKT/PKB pathways
Source: FEBS Open Bio. 2020 Nov 19;10(12):2541–52. doi: 10.1002/2211-5463.12987 (PMC7714072; doi:10.1002/2211-5463.12987)

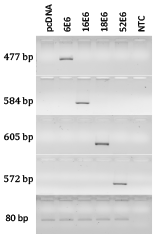

Supplement: Supplementary file 1 — Fig S1. Amplification of E6 genes was evaluated in cells transfected with different E6 constructs (HPV‐6 E6, HPV‐16 E6, HPV‐18 E6, and HPV‐52 E6) by end‐point PCR. Cells transfected with empty vector (Control cells) and a non‐template control (NTC) were included in each PCR reaction. As a loading control, a fragment of the beta‐globin gene was amplified. PCR products were resolved by electrophoresis in a 1% agarose gels stained with SYBR green. [file FEB4-10-2541-s001.tif]

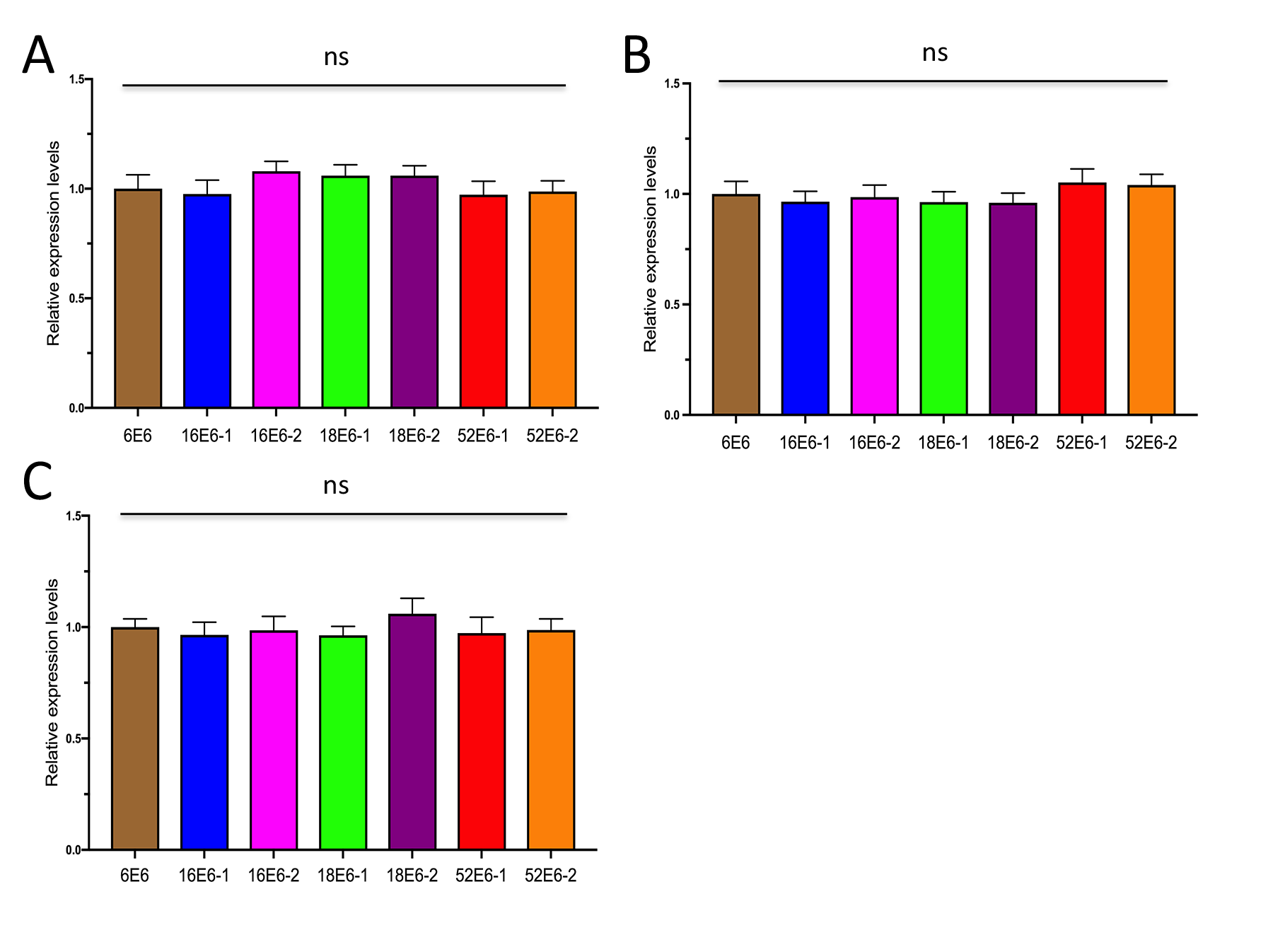

Supplement: Supplementary file 2 — Fig S2. Expression level of E6 mRNA in HEK293 (A), MCF7 (B), and HaCat (C) cells transfected with different E6 constructs (HPV‐6 E6, HPV‐16 E6, HPV‐18 E6, and HPV‐52 E6) was evaluated by RT‐qPCR. Expression levels of cells transfected with 16E6, 18E6, and 52E6 were compared with cells transfected with 6E6; at least two different clones obtained after geneticin selection were evaluated. Data of gene of interest were normalized with respect to GAPDH expression. Values represent mean ± SD of at least three experiments. Differences between groups were compared by one‐way ANOVA with Dunnett’s post hoc test (*p> 0.05; ns: no significance). [file FEB4-10-2541-s002.tif]

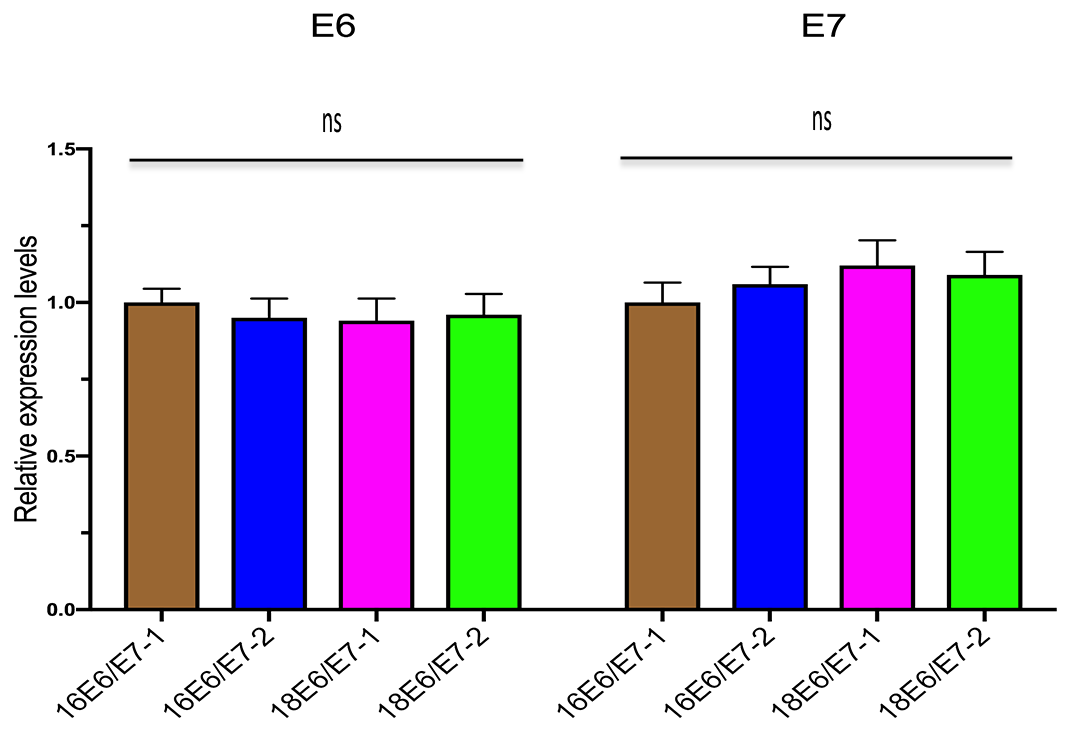

Supplement: Supplementary file 3 — Fig S3. Expression levels of E6 and E7 genes in HEK293 cells transfected with different E6/E7 constructs were evaluated by RT‐qPCR. Expression levels of E6 and E7 genes were compared between cells transfected with 16E6/E7 and 18E6/E7; at least two different clones obtained after geneticin selection were evaluated. Data of gene of interest were normalized with respect to GAPDH expression. Values represent mean ± SD of at least three experiments. Differences between groups were compared by one‐way ANOVA with Dunnett’s post hoc test (*p> 0.05; ns: no significance). [file FEB4-10-2541-s003.tif]
