# Supplementary material for: The high‐risk HPV E6 proteins modify the activity of the eIF4E protein via the MEK/ERK and AKT/PKB pathways
Source: FEBS Open Bio. 2020 Nov 19;10(12):2541–52. doi: 10.1002/2211-5463.12987 (PMC7714072; doi:10.1002/2211-5463.12987)
Supplement: Supplementary file 4 — Table S1. Primers sequences employed for end‐point PCR amplification. Table S2. Primer sequences for evaluation of gene expression by real‐time PCR. [file FEB4-10-2541-s004.docx]

SUPPORTING INFORMATION

Supplementary table 1.Primers sequences employed for end-point PCR amplification

| Genes | (5’-3’) | Position at 5´end | Position at 3´end |
| --- | --- | --- | --- |
| 6E6 | F - TTTAGCAAACGAGGCATT  R - GTTGCAGGTCTAATACAATAT | 7998 | 477 |
| 16E6 | F - CCGGTTAGTATAAAAGCAGAC  R - AATGTAGGTGTATCTCCATGCA | 57 | 584 |
| 18E6 | F - GTGAGAAACACACCACAATAC  R - CCTTAGGTCCATGCATACTTAAT | 83 | 605 |
| 52E6 | F - CACAGTGTAGCTAACGCACG  R - GTTGCTTTGTCTCCACGCATG | 79 | 572 |
| 16E6/E7 | F - CCGGTTAGTATAAAAGCAGAC  R - ATTGGTACCTGCAGGATCAGCCAT | 57 | 888 |
| 18E6/E7 | F - GTGAGAAACACACCACAATAC  R - CTTCTGGATCAGCCATTGTTG | 83 | 929 |

Suplementary table 2. Primer sequences for evaluation of gene expression by real-time PCR

| Gene | (5’-3’) | Amplified DNA fragment |
| --- | --- | --- |
| GAPDH | F - GAAGGTCGGAGTCAACGGATTT  R - ATGGGTGGAATCATATTGGAAC | 147bp |
| 6E6 | F - CGGTTYATAAAGCTAAATTGTACGT  R - AGGGTAACATGTCTTCCATGCA | 78bp |
| 16E6 | F - TTGCAGATCATCAAGAACACGTAGA  R - AATGTAGGTGTATCTCCATGCA | 64bp |
| 18E6 | F - AGAGGCCAGTGCCATTCGT  R - GTTTCTCTGCGTCGTTGGAGT | 64bp |
| 52E6 | F - GACATGTTAATGCAAACAAGCGAT  R - CATGACGTTACACTTGGGTCACA | 104bp |
| 16E7 | F - GATTTGCAACCAGAGACAACTG  R - CCAGCTGGACCATCTATTTCAT | 89bp |
| 18E7 | F - AATTCCGGTTGACCTTCTATGT  R - TCGGGCTGGTAAATGTTGAT | 97bp |
| CCND1 | F - AACTACCTGGACCGCTTCCT  R - CCACTTGAGCTTGTTCACCA | 203bp |
| eIF4E | F - CAGAGACGAAGTGACCTCGAT  R - GCTATCTTATCACCTTTAGCTCTAA | 130bp |
| ODC1 | F - GAAGATGAGTCGAGTGAGCAGA  R - CAACAATCCGATCGAGGCCAT | 186bp |
